# Sensitivity of a helical diode array device to delivery errors in IMRT treatment and establishment of tolerance level for pretreatment QA

**DOI:** 10.1120/jacmp.v13i1.3660

**Published:** 2012-01-05

**Authors:** Feliciano García‐Vicente, Virginia Fernández, Rocío Bermüdez, Alberto Gómez, Leopoldo Pérez, Almudena Zapatero, Juan J. Torres

**Affiliations:** ^1^ Department of Radiation Oncology and Medical Physics Hospital Universitario de La Princesa, Instituto de Investigación Sanitaria la Princesa Madrid 28006 Spain

**Keywords:** IMRT quality assurance, dose‐volume histograms, helical array, diodes

## Abstract

The aim of this study is to determine the gantry angle and multileaf collimator (MLC) gap error‐detection threshold of a diode helical array with an inserted microionization chamber in order to use this device for the pretreatment quality assurance (QA) of intensity‐modulated radiation therapy (IMRT) treatments. Implications on the dose‐volume histograms (DVHs) of the patient treatments will also be considered for the establishment of a QA protocol with a reasonable tolerance level. Three dynamic IMRT HN (head and neck) and prostate treatments were studied. Random and systematic variations of gantry angle and systematic errors in MLC gap width of the clinical treatments were analyzed in order to establish the detection sensitivity of the array. The associated clinical significance was studied introducing the same errors in the treatment plan based on the patients' computed tomography (CT) and calculating the corresponding DVHs. The Gamma (3%/3 mm) presented a 4% variation in failure rate for a rotation error of 1° for both types of treatment. Both systematic and random errors in gantry rotation angle have little effect on the patients' DVHs. MLC gap width errors of 1 mm and 2 mm in the prostate treatments imply a mean variation in isocenter‐measured absorbed dose of 2.1% and 4.1%, respectively. In the case of HN, these errors entail a change in measured isocenter dose of 4.7% and 8.6%, respectively. The variation observed in the DVHs of the patients was, basically, a global displacement of the curves proportional to the isocenter dose variation caused by the gap width error. According to the array sensitivity to the analyzed errors and its implication in patient DVHs, a tolerance of 95% point passing rate for the gamma criterion 3%/2 mm and an agreement of 2% in isocenter absolute dose have been established as tolerance criteria for our pretreatment IMRT QA protocol.

PACS number: 87.56.Fc

## I. INTRODUCTION

The introduction of IMRT treatments has required the use of dosimetric systems for QA. This pretreatment verification process was initially based on ionization chamber measurements, together with film dosimetry. The complexity and inefficiency of this procedure led to the use of systems that provide real‐time measurements based either on silicon portal dosimetry or on two‐ or three‐dimensional arrays of ionization chambers or diodes. Due to characteristics of some of these devices, the verification is usually done at zero degree gantry angle. This could lead to the overlooking of some potential errors as the result of the gantry rotation and the MLC positioning. Systems with detectors' configuration that allows for three‐dimensional dose verifications have recently become available. These systems could be used for step‐and‐shoot or sliding window IMRT treatments, as well as volumetric‐modulated arc therapy (VMAT) and tomotherapy.

Several authors have reported the implementation of gantry‐mounted 2D arrays^(^ ^1^ ^)^ and EPIDs^(^ ^2^ ^–^ ^3^ ^)^ for 3D pretreatment IMRT QA. The energy fluence readings provided by these systems can be combined with dose calculations obtained with suitable algorithms or special software so as to compare planned dose distributions with measured dose distributions.

Delta4 system (ScandiDos AB, Uppsala, Sweden) was the first commercial 3D array and has already been described by several authors.^(^ ^4^ ^–^ ^5^ ^)^ It consists of two two‐dimensional diode arrays embedded in a cylindrical phantom. It performs absolute and relative dose measurements, as well as integrated or per control point dose measurements. The usefulness of this device for different types of treatments has already been studied by several authors.^(^ ^5^ ^–^ ^6^ ^)^

The device analyzed in this work is commercially named ArcCHECK (Sun Nuclear, Melbourne, FL). It is made up of 1386 n‐type diodes embedded in a cylindrical PMMA phantom and distributed in a helical arrangement. As with the above‐mentioned system, it provides a correction for sensitivity differences in the diodes and variation in angular response with beam incident angle. The characteristics concerning the design, response, calibration, and sensitivity of this device have been reported previously.^(^ ^7^ ^–^ ^8^ ^)^ However, no studies have been published so far that assess the sensitivity for detecting positioning errors of the gantry and MLC, and the direct clinical implication of these errors on patient DVHs. The aim of this study is to evaluate the error‐detection threshold of this array for the pretreatment IMRT QAs. Implications on the DVHs of the patient treatments will also be considered for establishing a QA protocol with reasonable tolerance levels.

## II. MATERIALS AND METHODS

### A. Description and commissioning of ArcCHECK

**Figure 1:**
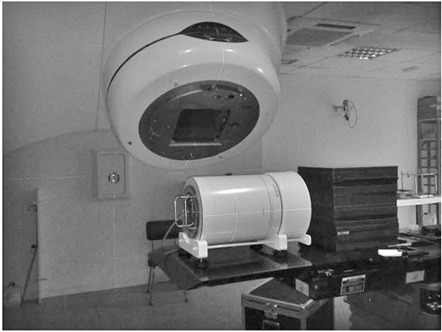
Picture of the experimental setup. This setup includes a stand made of carbon fiber in order to avoid the influence of the treatment couch and a microchamber placed inside an acrylic insert for isocenter dose measurement.

The ArcCHECK device and the setup are shown in Fig. 1. The system consists of a $0.8\;\times \;0.8\;{\text{mm}}^{2}$ n‐type diode array, which is embedded in a cylindrical PMMA phantom in a helical geometric arrangement with a separation of 10 mm between adjacent detectors. The positions of diodes on adjacent rings are rotationally offset by 1 cm to form a spiral configuration. Each diode has an acrylic buildup of 3 cm, which is equivalent to 3.5 cm of water. The device has a detection length of 21 cm and an array diameter of 21 cm. The phantom can accommodate an insert of 15 cm of diameter, which can house ionization chambers, as well as different heterogeneities. We made measurement with a PMMA insert that housed a PinPoint 31014 microchamber (PTW, Freiburg, Germany) to determine the isocenter dose. Our protocol includes absolute isocenter dose measurement in order to have an experimental absolute dose value obtained with an ionization chamber in a representative point of the target volume. The device also includes a stand and alignment marks.

We evaluated the basic performance of the device, including reproducibility, linearity, and background prior to using it in the clinic. This performance evaluation included a comparison with measurements using film dosimetry and ion chamber in a pelvic and HN phantom. Our former IMRT QA method, using film and ion chamber, showed a mean gamma pass rate of 98.7% using 3%/3 mm criteria for a set of 180 prostate patients, with the film measurements normalized to the isocenter dose. The new ArcCHECK system showed a mean gamma pass rate of 99.4% for 20 patients (15 prostate and 5 HN). This analysis included 10% dose threshold, normalization to the maximum dose in the detector plane, and a two‐dimensional dose tolerance agreement (DTA) search. Points receiving a dose value under the 10% of the maximum dose in each map were excluded in order to reduce the favorable bias for the inclusion of low dose points.

The software provided with the measuring device, MapCHECK v. 5.02 (Sun Nuclear, Melbourne, FL), was used for every comparison of dose distributions.

### B. IMRT planning and dose calculation

This study was carried out for the two most widely spread IMRT treatments: prostate and HN. Three dynamic IMRT (sliding window, 320 control points per beam), HN, and prostate treatments were analyzed. These treatment plans were generated to satisfy the normal tissue and target coverage criterion established at our institution which is given in Table 1. For prostate IMRT, the treatment was based on a geometric disposition of five 15 MV beams with angles of 260°, 324°, 36°, 100°, and 180°. For HN treatments, a geometric structure of seven equally spaced posterior 6 MV beams was used, with angles of 180°, 150°, 120°, 90°, 270°, 240°, and 210°. Depending on the PTV size, three or five of these beams required splitting, due to the limitations imposed by the length of the MLC employed (Millennium 80, Varian Medical Systems, Palo Alto, CA). Each treatment plan was recalculated on the CT image of the ArcCHECK phantom at 1 mm×1 mm×1 mm resolution, and the resultant three‐dimensional dose matrices were exported via DICOM to the computer in which dosimetric comparisons are carried out. These matrices are the base for comparison with the different measures obtained with the helical array. The XIO 4.5 (Elekta AB, Stockholm, Sweden) treatment planning system (TPS), which employed convolution/superposition algorithm with a pixel by pixel heterogeneity correction, was used. All patients' plans were calculated at 2 mm×2 mm×2 mm resolution.

**Table 1:** Summary of dose prescription for the cases analyzed in this study. In this notation VXX < YY% means that the maximum volume that receives XX Gy should be YY%.

*Head and Neck*		
PTV66		95%>66 Gy V72.4<20%
PTV54		95%>54 Gy V59.4<20%
Brain stem		$D_{\text{max}}<54\;\text{Gy}$
Parotids		$D_{\text{mean}}<26\;\text{Gy}$
Eyes		$D_{\text{max}}<50\;\text{Gy}$
Spinal cord		$D_{\text{max}}<50\;\text{Gy}$
Chiasm		$D_{\text{max}}<50\;\text{Gy}$
Glottis		V50<66%
*Prostate*		
PTV		98%>76 Gy $D_{\text{max}}<85\;\text{Gy}$
Rectum		$D_{\text{mean}}<48\;\text{Gy}$ V72<25% V60<40%
Bladder		V70<30%
Fem. heads		V52<5%

The treatment plans were recalculated after introducing the simulated errors and the resultant DVHs were studied. These simulated errors consisted of random and systematic variations of 1° and 2° in the gantry angle, and systematic errors of 1.0 mm and 2.0 mm outwards in the gap width of the MLC segments (distance projected at isocenter between opposing leaves). MLC gap random errors were not simulated because, when considering MLC positioning, these kinds of errors have always been less dosimetric significance than MLC systematic errors due to compensation effects. Inwards errors were not simulated in order to avoid mechanical collisions between opposing leaves. To simulate systematic gantry errors, the gantry angle of all beams was modified $+1^{0}$ in one case and $+2^{0}$ in the other. In order to simulate random errors, the gantry angle for each beam was randomly modified $+1^{0}$ or $-1^{0}$, in one case and $+2^{0}$ or $-2^{0}$ in the other. We analyzed two types of MLC gap errors: those affecting every pair of opposing leaves, or just a single pair. The error in the MLC leaves was introduced by modifying the MLC files in the TPS using an in‐house software. We selected these linear accelerator (linac) parameters because they are probably the most error sensitive in such kind of treatments, and the error in gap size of the MLC is considered the most critical parameter regarding the accuracy of the dose output in dynamic IMRT treatments.^(^ ^9^ ^)^

### C. IMRT plan delivery and array measurement

In order to avoid the influence of the treatment couch with some beam incidences, the experimental setup includes a stand made of carbon fiber. The device was placed in this stand in such a way that the linac isocenter matched with the alignment marks and with the longitudinal axis of the device in the gun‐target direction.

As previously stated, several treatments were created and measured for each patient. The first one corresponded to the treatment without errors (clinical treatment), while the rest (nonclinical treatments) included the mentioned errors.

The treatment was delivered with a Clinac 2100 CD (Varian Medical Systems, Palo Alto, CA), equipped with an 80‐leaf MLC with 1 cm leaf width projected at the isocenter. Prior to each measurement, a background measurement was performed for noise reduction. Although the system measures absolute dose, we chose to use ion chamber (0.015 cc) placed at the axis of the phantom to measure the absolute dose from the treatment fields at isocenter and, as well, to account for the daily output variations of the accelerator by measuring dose from standard fields. We performed a dose measurement in a 10 cm×10 cm beam with the phantom aligned with the machine's isocenter and with the number of monitor units (MU) necessary to obtain a dose value of 100 cGy at the phantom's isocenter when the machine is perfectly calibrated (123 MU for 15 MV, 152 MU for 6 MV). Dividing the dose readings corresponding to the IMRT beams by this reference dose value and multiplying by 100, we obtained the isocenter dose with the output variation already taken into account.

The measured and calculated doses were compared using gamma analysis^(^ ^10^ ^)^ with DTA tolerance of 3 mm and 3% in dose difference (%ΔD) always in relative dose. The dose difference was computed normalizing to the maximum dose of the entire cylindrical plane, and DTA was calculated with a two‐dimensional search. These were the criteria for gamma comparison in our former QA verification protocol. For this study, we repeated the analysis, with DTA criterion of 1 mm and the %ΔD of 2% to obtain the optimal metric that gave the maximum difference in percent of detectors passing the gamma criteria between the original plan and the plan with simulated errors. Absolute dose gamma analysis has not been performed because it would require a daily absolute dose calibration of the ArcCHECK device. As has been previously stated, absolute isocenter dose measurement with ionization chamber is metrologically and geometrically more adequate, and does not involve an increase in complexity or in measurement time.

## III. RESULTS

**Figure 2:**
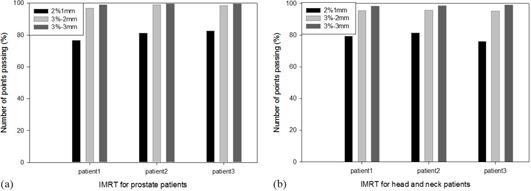
Histogram of the relative number of diodes passing the gamma evaluation criteria. The insert shows the criteria used.

The agreement between calculated and measured dose values for three prostate and three head and neck treatments without introduced errors was good. On average, 99.1% of the diodes (range 98.3%–100%) met the criterion of 3%/3 mm, and 96.7% (range 95.5%–98.6%) met the criterion 3%/2 mm. Remarkable differences were not observed between prostate treatments (low degree of modulation) and HN treatments (high degree of modulation) (Fig. 2). The gamma criteria 2%/1 mm resulted in a very low fraction of fulfilling points (79.5%) and very high dispersion (max 82.7%, min 75.9%) so as to be established as a basis for a QA protocol. The disagreement between calculated and measured isocenter dose was below 0.5% in every analyzed treatment.

**Figure 3:**
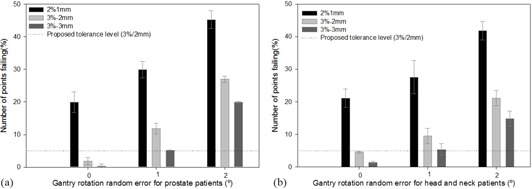
Histogram of the relative number of diodes which did not satisfy the different tolerance criteria for the prostate and HN plans as a function of gantry rotation error. The error bars show the maximum interpatient variation.

As can be observed in Fig. 3, the threshold for error detection regarding gantry rotation is about one degree for both kinds of treatments, considering the 3%/3 mm criterion as well as the 3%/2 mm criterion. The criterion 3%/3 mm presents a 4% variation in failure rate for a rotation error of one degree for both types of treatment. The criterion 3%/2 mm shows a different behavior for each of the analyzed treatments, yielding a mean variation of 10% for prostate and 5% for HN. Therefore, considering the criterion 3%/3mm with a 98% points pass rate limit or the criterion 3%/2 mm with a 95% points pass rate limit, we would detect an error in gantry rotation up to 1°. Taking into account that the criterion 3%/2mm shows higher sensitivity to gantry angle error detection than the criterion 3%/3mm, we have selected the former for our IMRT QA protocol.

Considering the gap width variation in all leaf pairs within the field, the gamma analysis in relative dose regarding both the criterion 3%/3 mm and the criterion 3%/2 mm shows, with both gap width errors and in both IMRT treatments, similar results to those obtained in the treatments without error. Therefore, these errors would not be detected unless a stricter gamma criterion was used, leading us to lower the criterion to 2%/1 mm so that the system was able to detect a 2 mm gap error. Nevertheless, as has already been stated, this last option would not result in a feasible QA protocol. This is due to the intrinsic calculation errors coming from the TPS. These errors would yield gamma results providing insufficient information with which to validate a treatment. Besides, this criterion yields an erratic behavior of the gamma pass rate, showing very high patient‐to‐patient deviations (gamma 2%/1 mm, max 87.1%–min 67.3%). Respectively, gap errors of 1 mm and 2 mm imply a mean variation in isocenter measured absorbed dose of 2.1% (max 2.6%, min 1.9%) and 4.1% (max 4.9%, min 3.7%). In the case of HN, the same errors entail a change in measured isocenter dose of 4.7% (max 5.1%, min 4.3%) and 8.6% (max 9.1%, min 7.7%), respectively. This effect has previously been described by other authors.^(^ ^9^ ^)^ Basically, it is due to the fact that the dose error depends on both the error in gap width and the treatment mean gap width, which is much smaller in high degree modulation treatments (HN) than in low degree modulation treatments (prostate). Therefore, it would be sufficient to perform a dose measurement at isocenter to notice a 1 mm gap error in both prostate and HN treatments.

**Figure 4:**
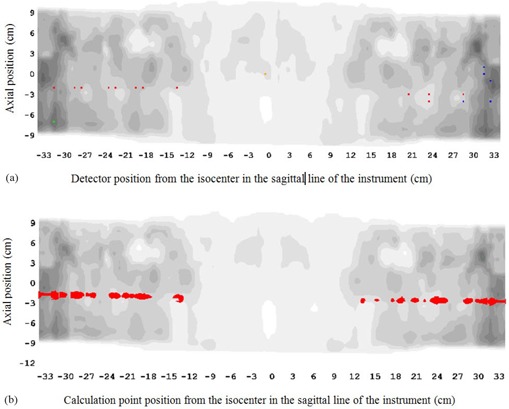
Comparison between calculated (without error) and measured (with 2 mm gap error) dose map (a) along the cylindrical measurement surface for the HN plan; (b) the same comparison between calculated dose distribution with and without gap error. The line depicted by the red dots corresponds to the detector circle receiving the dose coming from the leaf pair with the gap error. The red and blue dots represent the out of tolerance diodes with a dose superior and inferior to the TPS results, respectively. The blue dots also appear in the comparison of the clinical plan and are probably due to TPS calculation errors.

We also simulated the same gap errors (1 mm and 2 mm outwards) but only in one pair of leaves. In this case the isocenter dose measurement did not detect the error because the leaves with gap error were two centimeters away from the isocenter. In Fig. 4(a) we show the comparison between calculated (without error) and measured (with 2 mm gap error) dose map along the cylindrical measurement surface for the HN plan. Also, in Fig. 4(b) we show the same comparison, but between calculated dose distribution with and without gap error. The red and blue dots represent the out of tolerance diodes with a dose 3% superior and inferior to the TPS results, respectively. The blue dots also appear in the comparison of the clinical plan and are probably due to TPS calculation errors. The green dot represents the normalization point. As we can see, the device detects the error (red points in the horizontal line corresponding to the leaf pair with gap error) and matches the position of the dose discrepancy calculated for the TPS. In the case of prostate, the analysis did not detect the error because the effect is less than 3% of the normalization (maximum) dose.

**Figure 5:**
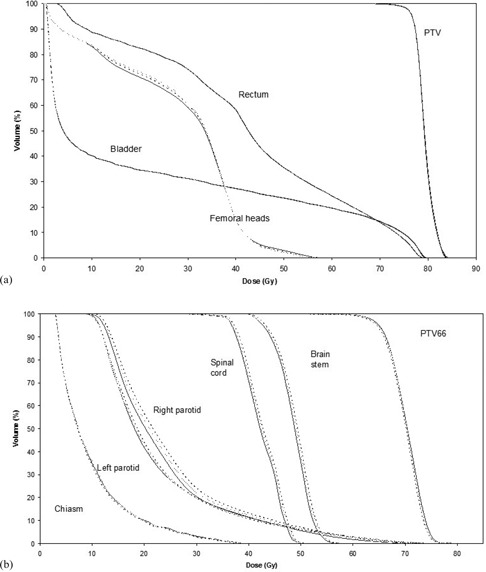
Dose‐volume histograms for the prostate (a) and HN (b) patients with the highest error influence on DVH, according to gantry angle error. Continuous line: no gantry error; dashed line: 1° gantry error; dotted line: 2° gantry error. Some of the structures in the HN DVH are not shown for clarity purpose.

The effects of the gantry angle errors on the DVHs of the different treatments considered are presented in Figs. 5(a) and 5(b). The study shows that systematic and random errors in gantry rotation angle of one degree have little effect on the DVHs and only in one HN patient. Figure 5 shows the differences encountered in DVHs in the patient with the highest error influence on DVH for each kind of treatment (Fig. 5(a) for prostate, Fig. 5(b) for HN). Therefore, when considering the gantry rotation errors, a gamma (3%/2 mm) pass rate of 95% of the diode measurement points seems to be an adequate threshold point.

**Figure 6:**
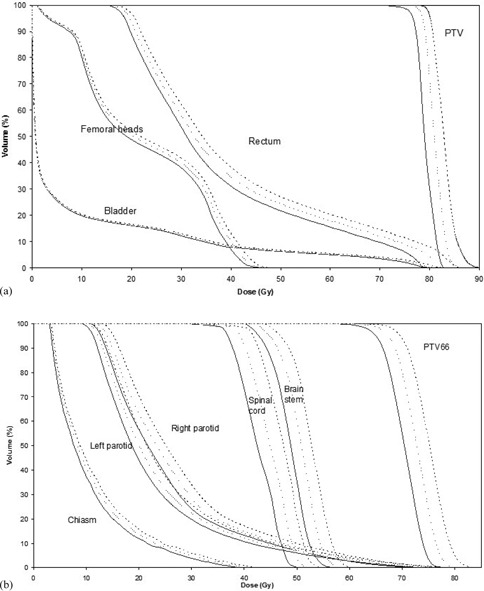
Dose‐volume histogram for the prostate (a) and HN (b) patients with the highest error influence on DVH, according to gap error. Continuous line: no gap error; dashed line: 1 mm gap error; dotted line: 2 mm gap error. Some of the structures in the HN DVH are not shown for clarity purpose.

Our study shows that if the positive gap width error affects all pairs of leaves uniformly, then the result is an increase of the absorbed dose in PTVs and OARs for both prostate and HN treatments (Fig. 6): the shape of the error‐free and error‐introduced DVH curves are very similar. The mean and maximum PTV and OARs doses increase proportionally to the gap width error (Table 2). Although negative gap width errors in the MLC leaves have not been considered to avoid mechanical collisions between opposing leaves, the effect would be a decrease in dose of approximately the same magnitude as seen with the positive gap width error.^(^ ^9^ ^)^ The similarity in DVH shape agrees well with the relative dose measurements because no gamma pass rate differences were observed between error‐free and error‐introduced treatments. This means, relative dose distributions are alike and, when this happens, the curves on the corresponding DVHs are identical, except for a scale factor. The exact value of this scale factor depends on the particular morphology of PTV–OAR, but we have found that approximately all the doses increase a mean of 4.5% per mm of gap width error in HN treatments (3.3%/mm for PTVs and 4.9%/mm for OARs) and 2% per mm of gap width error in prostate treatments (Table 2). These results agree well with the differences found in measured isocenter dose: 4.7% and 2.1 %, respectively. If the error only affects some pair of leaves, then its effect on DVH depends on the magnitude of the gap error and on the exact position of the malfunctioning pair of leaves regarding to the PTV and OAR.

**Table 2:** Ratios of error free to 1 mm gap error and 1 mm gap error to 2 mm gap error for calculated mean and maximum doses. The table shows mean values for all the analyzed patients for prostate and HN treatments. Note that the ratios are approximately constant for each kind of treatment and for each analyzed volume. Interpatient variation was not significant.

*Volume*	*Mean Dose*		*Maximum Dose*	
	Clinical/Gap+1 mm	Gap+1 mm/Gap+2 mm	Clinical/Gap+1 mm	Gap+1 mm/Gap+2 mm
		*Head and Neck Patients*		
PTV66	0.97	0.97	0.97	0.96
PTV54	0.97	0.97	0.97	0.96
Brain stem	0.96	0.96	0.97	0.97
L. Parotid	0.94	0.94	0.97	0.98
R. Parotid	0.93	0.94	0.95	0.95
L. Eye	0.94	0.94	0.95	0.95
R. Eye	0.94	0.95	0.95	0.95
Spinal cord	0.95	0.95	0.94	0.97
Chiasm	0.94	0.94	0.96	0.96
Glottis	0.95	0.96	0.96	0.96
		*Prostate Patients*		
PTV	0.98	0.98	0.97	0.99
Rectum	0.97	0.97	0.97	0.98
Bladder	0.98	0.98	0.97	0.99
Fem. heads	0.96	0.98	0.96	0.98

Taken into account all these results, we set a minimum gamma (3%/2mm) pass rate of 95% of the measurement points, together with an isocenter dose agreement of 2% as tolerance criteria. In addition, a visual inspection of the comparison of calculated and measured dose map along the cylindrical measurement surface is mandatory in order to detect gap error patterns (Fig. 4(a) horizontal red or blue lines).

Since the implementation of the presented QA protocol, 59 IMRT treatments have been performed in our center. High agreement has been observed concerning isocenter absolute dose (0.1% mean, 0.7% max.) and a mean gamma 3%/2mm value of 95.8% (range 95.1%–99.5%) has been obtained.

## IV. DISCUSSION

The establishment of a protocol for IMRT pretreatment verification directly related to clinical impact through PTV coverage parameters or OARs sparing is the ideal goal of the IMRT QA protocols.^(^ ^11^ ^–^ ^13^ ^)^ This is due to the fact that clinical outcome depends ultimately on these clinical parameters and, given the complexity of IMRT dose distributions, it is not as straightforward to transfer dosimetric discrepancies in measured points or planes to parameters in DVHs as it is in nonmodulated beams. The achievement of this aim has been restricted so far by the limitations of standard measuring devices^(^ ^14^ ^–^ ^18^ ^)^ stemming from their complexity, inefficiency, or design.

The agreement between calculated and measured dose distributions, as well as the sensitivity to errors in the gantry position, are similar to those found by Létourneau et al.^(^ ^7^ ^)^ for prostate and HN treatments employing the VMAT technique using a prototype of the studied QA device. Here, we show that 3%/2 mm is the optimal criterion for gamma analysis using gantry angle and gap width error analyses. While in the Létourneau study possible clinical implications of these errors are discussed, the reader is referred to subsequent further analyses. Here, we have completed the analysis suggested by these authors using stationary beam IMRT (sliding window) technique, and shown that the influence of one degree in the gantry angle error (systematic or random) is negligible regarding PTV coverage or organ sparing for both types of analyzed treatments. We found that a threshold level of 95% pass rate using a gamma analysis (3%/2 mm) to be optimal for the detection of gantry angle error.

To our knowledge, this is the first work that analyses the sensitivity of an IMRT QA device to errors in the gap width of the MLC segments and its impact on the corresponding DVHs. LoSasso et al.^(^ ^9^ ^)^ reported that the gap width error is one of the most important parameters in dynamic IMRT, noting that the smaller the average gap size, the greater its importance. The results of our study confirm the conclusions reached by LoSasso et al. The dosimetric impact of the gap width error depends not only on the error in gap size, but also on the mean gap size of the treatment. That is, unlike the case of the gantry rotation, it depends on the modulation degree required for the specific type of treatment. Moreover, it is established that the most effective way of gap error detection and quantification is the absolute dose measurement.^(^ ^9^ ^)^ Gap errors that would lead to variations in absolute dose of 9% would not be detected by means of relative dose gamma analysis. This result seems reasonable, as this error in dose comes from a variation in MLC leaves position of only 1 mm. Unless an analysis with geometric requirement of such order is carried out, this error cannot be detected in relative dose if the gap error is the same in all the pairs of leaves included in the treatment field. This situation can arise due to an incorrect calibration of the MLC or an incorrect modeling of the TPS. In order to detect this kind of error, ionization chamber absolute dose measurement in one or more points and/or array measurement and gamma analysis in absolute dose mode are necessary. As previously explained, considering the kind of QA protocol we are dealing with, the type of gamma analysis required would not be useful in the IMRT pretreatment verification, due to, among other factors, the intrinsic errors coming from the TPS. If the gap error is limited to some leaf pairs included in the treatment field, it will be detected by the device only if the dose effect is higher than the evaluation dose criteria, since the array detectors that correspond with the malfunctioning leaves will measure a higher/lower dose (from 2%/mm to 5%/mm) than the calculated dose. This error will be detected by the ionization chamber only if the position of the malfunctioning leaves corresponds to the chamber position. This error can occur due to motor malfunctioning, count losses of the motor primary encoder, or mechanical wear of the drive nut of the leaf. This error occurs occasionally in the treatment units and can also be easily detected with the Picket Fence test proposed by LoSasso et al.^(^ ^9^ ^)^

Besides what has already been experimentally established, the simulation of the gap width errors in the TPS yields the expected results. The dosimetric impact regarding PTV coverage and OAR sparing depends on the mean gap size — that is, on the type of treatment, the gap width errors, and the position of the malfunctioning leaves. When the gap error affects all pairs of leaves included in the treatment field, the variation observed in the DVHs is, basically, a global displacement of the curves proportional to the isocenter dose variation caused by the error. This result supports the convenience of the measurement of isocenter dose because it is directly related to the expected errors in patient DVHs when the treatment has a global error in the MLC gap.

Accepting the radiotherapy treatment accuracy requirements^(^ ^19^ ^)^ and considering the uncertainties in the different steps of the radiotherapy process, Ahnesjö and Aspradakis^(^ ^20^ ^)^ outlined an optimal accuracy in dose calculation of about 2%. In our QA procedure, besides the TPS calculation, delivery errors are also included; nevertheless, a tolerance value of 2% between isocenter calculated and measured dose has been established as a conservative criterion. Our experience has proved that this limiting value is easily achievable, since it has never been exceeded in our set of more than 180 patients (mean 0.5%, standard deviation 0.5%).

In a recent study Nelms et al.^(^ ^13^ ^)^ concluded that the per‐patient IMRT QA based on per‐beam planar dosimetry and gamma analysis does not detect errors that may be clinically important regardless of the measurement system. They also suggested that a similar study should be done for IMRT QA based on composite dose and for a QA device of the same type as the one that has been analyzed in the present study. Our work, although due to its experimental methodology analyzes a small sample of patients, confirms that the combination of gamma analysis (3%/2 mm), measurement of absolute dose in the isocenter, and inspection of the distribution of errors in the measurement plane of the array, ensures that (for the combination of treatment hardware, software of the TPS, measurement & analysis system used, and for the types of errors simulated) errors are detected that could have clinical impact. This apparent disagreement is the result of the type of methodology employed. First, the fact that we have analyzed the composite plan avoids situations where false positives (hot or cold spots) could appear in per‐beam QA that could compensate/dilute in the composite treatment. Moreover, our results coincide for errors that affect the dose globally, such as the value of MLC leakage or gap. It is not possible to detect this kind of errors by relative gamma analysis. In any case it would be helpful to provide, as indicated by Nelms et al.,^(^ ^13^ ^)^ QA systems that compute accurately DVHs from QA device dose measurements.

Although, due to the experimental nature of this study which was limited to two specific types of treatment (prostate and HN) since the geometric arrangement of the beams, the levels of modulation, and the geometry of PTV‐OAR are very different, the generalization of the results to other pathologies seems reasonable.

## V. CONCLUSIONS

According to the array sensitivity to the analyzed errors and its implication in patient DVHs, an efficient QA procedure for IMRT pretreatment verification has been stated. A tolerance of 95% point passing rate for the gamma criterion 3%/2 mm and an agreement of 2% in isocenter dose have been established. In addition, in the absence of absolute dose‐based gamma analysis, we recommend the measurement of absolute dose at isocenter using an ion chamber because relative gamma analysis was not able to detect gap width error that is uniform across all MLC leaves. Moreover, this avoids the need for array recalibrations, and the time required to perform the whole QA process is not increased.

## ACKNOWLEDGMENTS

The authors would like to thank Dr. Ana Fernández for her suggestions on the manuscript. We would also like to thank the referees for their helpful comments, which have substantially improved this paper.
